# Phytochemicals targeting epidermal growth factor receptor (EGFR) for the prevention and treatment of HNSCC: A review

**DOI:** 10.1097/MD.0000000000034439

**Published:** 2023-10-06

**Authors:** Shaling Li, Yongdong Sun

**Affiliations:** a The Affiliated Hospital of Traditional Chinese Medicine of Southwest Medical University, Longmatan District, Luzhou City, Sichuan Province, China.

**Keywords:** epidermal growth factor receptor (EGFR), head and neck squamous cell carcinoma (HNSCC), phytochemicals

## Abstract

Head and neck squamous cell carcinoma (HNSCC) develops from the mucosal epithelium of the oral cavity, pharynx, and larynx, and is the most common malignancy of the head and neck, the incidence of which continues to rise. The epidermal growth factor receptor is thought to play a key role in the pathogenesis of HNSCC. Inhibition of epidermal growth factor receptor has been identified as an effective target for the treatment of HNSCC. Many phytochemicals have emerged as potential new drugs for the treatment of HNSCC. A systematic search was conducted for research articles published in PubMed, and Medline on relevant aspects. This review provides an overview of the available literature and reports highlighting the in vitro effects of phytochemicals on epidermal growth factor in various HNSCC cell models and in vivo in animal models and emphasizes the importance of epidermal growth factor as a current therapeutic target for HNSCC. Based on our review, we conclude that phytochemicals targeting the epidermal growth factor receptor are potentially effective candidates for the development of new drugs for the treatment of HNSCC. It provides an idea for further development and application of herbal medicines for cancer treatment.

## 1. Introduction

Head and neck squamous cell carcinoma (HNSCC) develops from the mucosal epithelium of the oral cavity, pharynx, and larynx and is the most common malignancy of the head and neck. HNSCC is the 6th most common cancer worldwide and its incidence continues to rise, with a projected 30% increase by 2030 (Global Cancer Observatory [GLOBOCAN]). Clinical treatment of HNSCC is currently dominated by surgery and anti-epidermal growth factor receptor (EGFR) monoclonal antibodies, but the adverse side effects and efficacy of anti-EGFR monoclonal antibody therapy are questionable and its use is accordingly limited.^[1]^ 1 In contrast, the significance of phytochemical agents in the treatment of HNSCC has been highlighted due to their tremendous efficacy and fewer adverse side effects.^[2,3]^ In recent years, rapid progress has been made in the pharmacological treatment of HNSCC.^[4]^ Inhibiting the early phases of carcinogenesis or boosting the effectiveness of standard chemotherapeutic treatments are 2 ways that chemicals of herbal origin can be used as chemo preventives and therapeutics, according to several experimental research.^[5,6]^ Phytochemicals are a good source of novel antitumor drugs.^[7,8]^ In vitro and in vivo studies have shown that Phytochemicals have a wide range of anticancer effects.^[9]^ Some of these phytochemicals have anti-inflammatory effects by limiting the release of cytokines from cells, such as IL-1, IL-6, IL-8, and IL-12. By inhibiting VEGFA or PI3/Threonine protein kinase (AKT), they in turn prevent cellular angiogenesis, reduce the proliferation of cancer cells, decrease the number of cancer stem cells, induce apoptosis, and regulate oncogene signaling pathways, which determine the growth and further development of cancer.^[10]^ Most significantly, the majority of phytochemicals function as antioxidants, preventing oxidative stress and potential cancerous effects on cells genetic material by scavenging oxygen-free radicals. Compounds of plant origin may impact cancer treatment by enhancing tumors’ susceptibility to chemotherapy and their in vitro cytotoxic effects, in addition to lowering cancer risk.

In the last decade, studies on molecular genetic mapping of HNSCC have revealed trends in therapeutic interventions. Ongoing work aims to integrate our knowledge of the biology and immunobiology of HNSCC to identify effective biomarkers that will enable more accurate and less toxic therapeutic options.^[11]^ Overexpression of EGFR is associated with advanced disease stages, underpinning the role of EGFR in tumor metastasis and invasion in patients with laryngeal cancer.^[12]^ Therefore, targeting EGFR is considered a reliable therapeutic strategy for patients with HNSCC. Numerous studies have shown that Phytochemicals, including Polyphenols, Flavonoids, and Terpenoids, can be used as therapeutic agents to achieve protective and therapeutic effects against HNSCC.^[13]^ Phytochemicals that target epidermal growth factor receptors are a potentially good source of new drugs for the treatment of HNSCC due to the benefits of phytochemicals and their reduced side effects. The development of effective therapeutic agents for HNSCC may benefit from research into the role of phytochemicals that target epidermal growth factor receptors in HNSCC.^[14,15]^ Throughout the investigation, we discovered that the majority of studies relied on cellular and animal models.^[16]^ The effectiveness of herbal chemical agents in HNSCC via EGFR should therefore be the subject of additional extensive clinical studies.

## 2. Methods

In this review, we searched PubMed/Medline for relevant articles published between 2003 and January 2023 using various combinations of keywords, including “EGFR,” “HNSCC,” “squamous cell carcinoma of the head and neck,” “Natural plant compounds,” and “Phytochemicals. “The articles we searched had to meet the following criteria; Focus on natural Phytochemicals that affect HNSCC via EGFR; Pharmacological studies performed in vitro in various cellular models and in vivo in animal models, and; English-language articles only; any papers that did not meet these criteria were excluded. It should be mentioned that because our search method was not flawless, some pertinent papers might not have been included in our study.

### 2.1. EGFR

**Figure 1.**
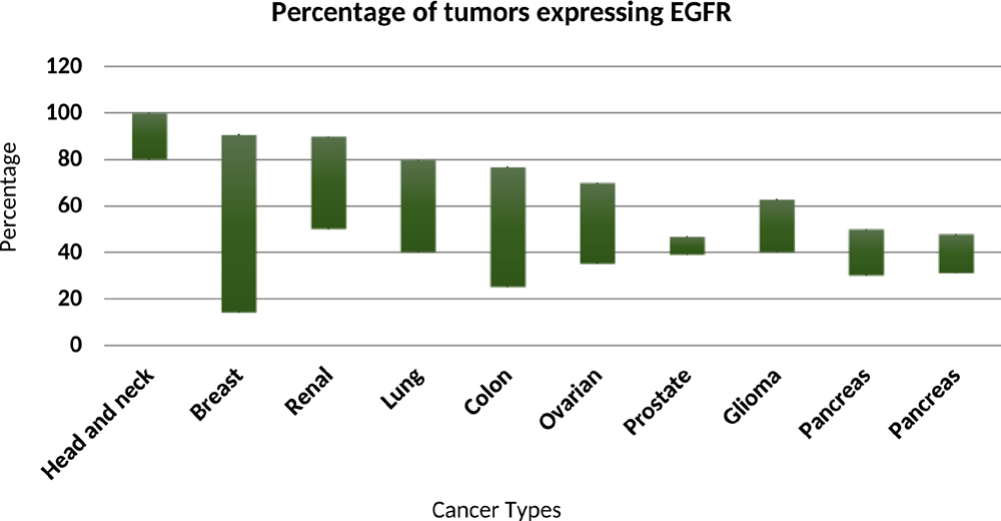
Frequency of EGFR expression in human tumors.^[29]^ EGFR = epidermal growth factor receptor^.^

The EGFR has been one of the most intensively studied cell surface receptors in the medical community, as its role in cancer biology, developmental biology, and tissue homeostasis has been recognized.^[17,18]^ EGFR consists of a polypeptide chain of 1186 amino acids and is expressed on the surface of most normal cells.^[19]^ It belongs to the HER receptor family and is a transmembrane receptor tyrosine kinase.^[20]^ Currently, 4 members of this family have been identified, including EGFR (HER1/erbB-1), HER2 (erbB-2/neu), HER3 (erbB-3), and HER4 (erbB-4).^[21]^ The 4 receptor genes and the currently known 11 ligands comprise a system of interactions between them with the ability to receive, process, and export information.^[22]^ The 4 members of the ErbB receptor family are among the approximately 60 receptor tyrosine kinases that have been identified in the human genome and are essential for several cell survival processes.^[23,24]^ The receptors of the ErbB family can be homo- or heterodimerized both compositionally and in response to ligands, leading to a great diversity of signaling.^[25]^ Among them, the EGFR plays a key function in epithelial cell physiology. It plays an important role in cell proliferation processes, differentiation, and apoptosis.^[26]^ In the case of cancer, uncontrolled autophosphorylation of EGFR occurs, leading to increased cell proliferation and decreased apoptosis, causing cancer promotion.^[27]^ As a widely studied biomarker, it is frequently mutated or overexpressed in different types of human cancers and remains a major therapeutic target in HNSCC, as its overexpression is detected in more than 90% of this cancer, and these alterations directly affect overall and progression-free survival rates^[17,28]^ (Fig. 1). Activation of EGFR in cancer cells can lead to the activation of several linear pathways, including mitogen-activated protein kinase, polypropylene cross-ester ethyl cross-ester, phosphatidylinositol-3-kinase/AKT, and the signal transducer and activator of transcription.^[30]^

In many common human tumor types, its members are also aberrantly activated by overexpression or mutation, making inhibitors of the EGFR a target for anticancer drug development.^[31]^ Epidermal growth factor receptor blockade is an attractive target for patients with HNSCC, and therapeutic targeting of EGFR with anti-EGFR monoclonal antibodies or kinase domain inhibitors, along with radiation therapy, remains a treatment option for patients with HNSCC.^[32,33]^ However, the efficacy of these therapies may be compromised, on the 1 hand, by the presence of preexisting genetic alterations in EGFR that make it resistant to EGFR blockade and, on the other hand, by the acquisition of secondary mutations under therapeutic pressure, which contribute to evasion of targeted therapy.^[34]^ Epidermal growth factor receptor status is increasingly recognized as a predictor of survival as well as response to chemotherapy in HNSCC.^[35,36]^ Published studies have shown that due to the multiple resistance mechanisms of EGFR inhibitors, different strategies are needed to improve the efficacy of EGFR inhibitors in HNSCC (Table 1).

**Table 1:**
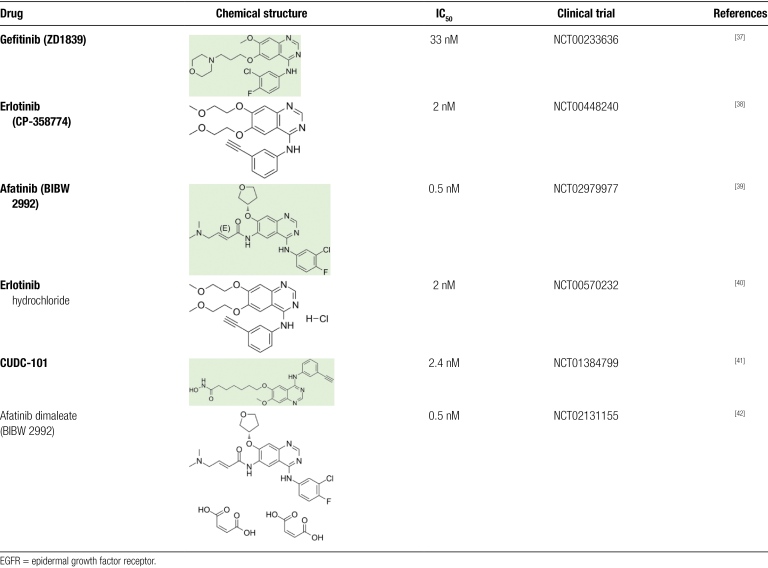
Clinical trial of EGFR inhibitors in high-risk head and neck cancer.

### 2.2. The role of phytochemicals in EGFR

Inhibition of the migration potential of HNSCC cells by phytochemicals involves; The inhibitory effect of phytochemicals on EGFR overexpression; The inhibitory effect of phytochemicals on extracellular signal-regulated kinase 1/2 (ERK1/2) protein activation and nuclear factor kappa (NF-κB) inactivation, and; Reversal of epithelial-mesenchymal transition (EMT).

The migratory capacity or metastatic potential of cells is an important factor in the high mortality rate of HNSCC and the difficulty of its treatment.^[43]^ NF-κB has been identified as an important regulator of cancer cell invasion, metastasis, and angiogenesis and is a downstream target of EGFR.^[44]^ Inhibition of the migration potential of head and neck squamous cell carcinoma cells by phytochemicals was associated with inhibition of ERK1/2, suggesting that the ERK1/2-NF-κB pathway may be involved in the effect of phytochemicals on HNSCC cell migration.^[45]^ The levels of this NF-B protein were decreased when phytochemicals were applied to head and neck squamous cell carcinoma cells.^[46]^ That is, NF-κB plays a role in determining the migration potential of HNSCC cells, and the inhibitory effect of phytochemicals on cell migration is mediated, at least in part, by the inactivation of NF-κB. Meanwhile, the EMT of cancer cells plays an important role in determining the metastatic potential of epithelial tumors. EMT can cause tumor cells to migrate and become invasive by affecting all stages involved, including invasion, infiltration, and extravasation.^[47]^ EMT also contributes to EGFR inhibitor resistance in cancers with EGFR mutations.

**Figure 2.**
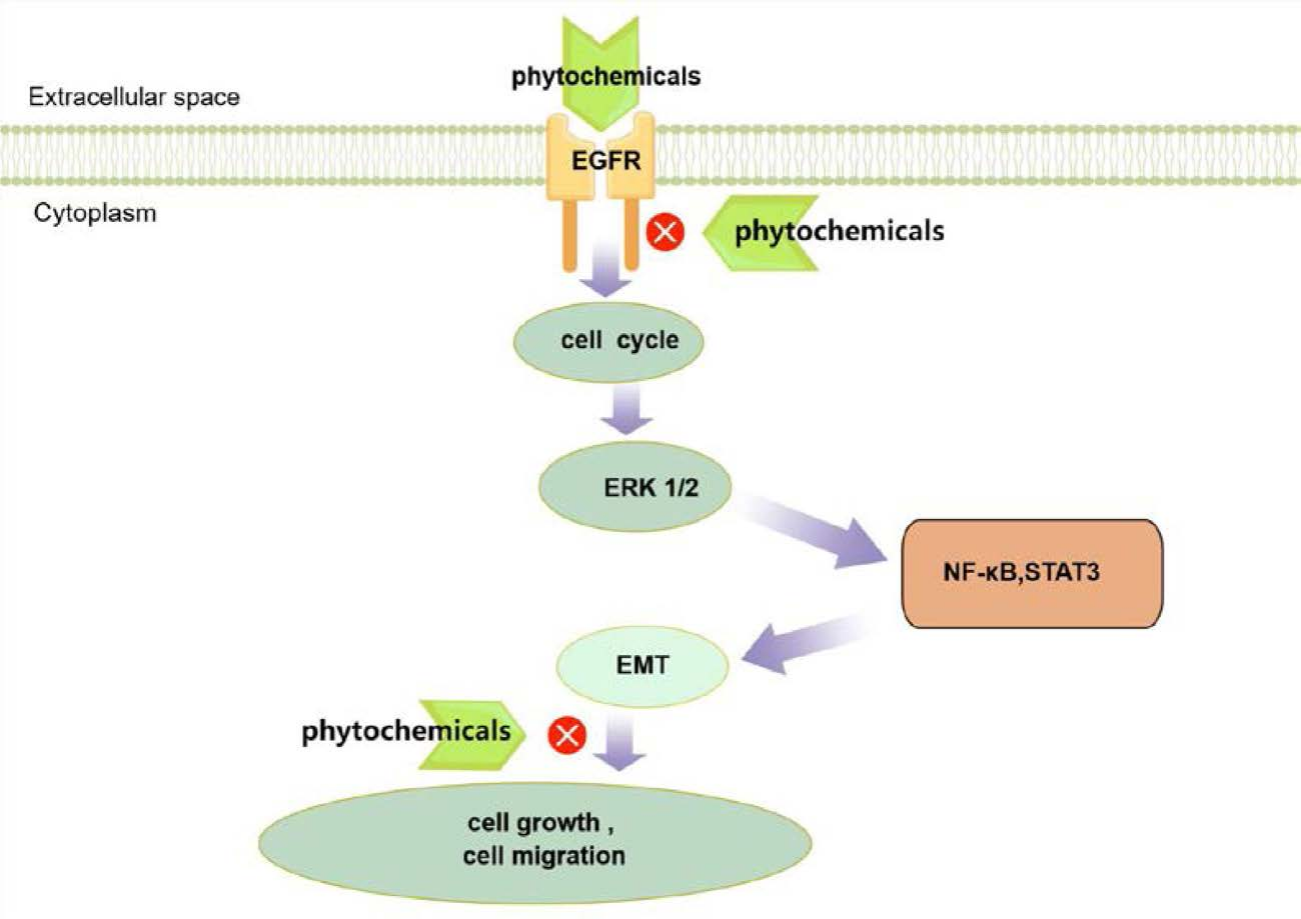
The role of phytochemicals in the EGFR signaling pathway (By Figdraw). EGFR = epidermal growth factor receptor.

Treatment of head and neck squamous cell carcinoma cells with phytochemicals resulted not only in the down-regulation of mesenchymal biomarkers but also the in reactivation of epithelial biomarkers.^[48]^ This suggests that phytochemicals can reverse the EMT process in HNSCC cells, and this reversal of NF-κB has been identified as a target in HNSCC cells, which has been identified as an important regulator of EMT in cancer cells^[49]^ (Fig. 2). These data suggest that inhibition of the migration potential of HNSCC cells by phytochemicals is involved. In summary, these data suggest that phytochemicals could be used as complementary and alternative drugs to prevent invasion and metastasis of HNSCC cells. However, further in vivo studies are needed to validate the therapeutic potential of these phytochemicals.

### 2.3. Phytochemicals targeting the EGFR in HNSCC

The slow development of treatments for HNSCC over the last 20 years has led to a reduction in 5-year survival rates.^[1]^ A growing body of evidence suggests that phytochemicals are promising leads against HNSCC and show potential as adjuvants to chemoradiotherapy for HNSCC.^[50,51]^ We collate data on a variety of leading phytochemicals that have shown promising results in the prevention and treatment of HNSCC. This literature and reports focus on the EGFR target of phytochemicals in different cellular models of HNSCC in vitro and animal models in vivo and elucidate the significance of EGFR as a current therapeutic target for HNSCC (Table 2).

**Table 2:**
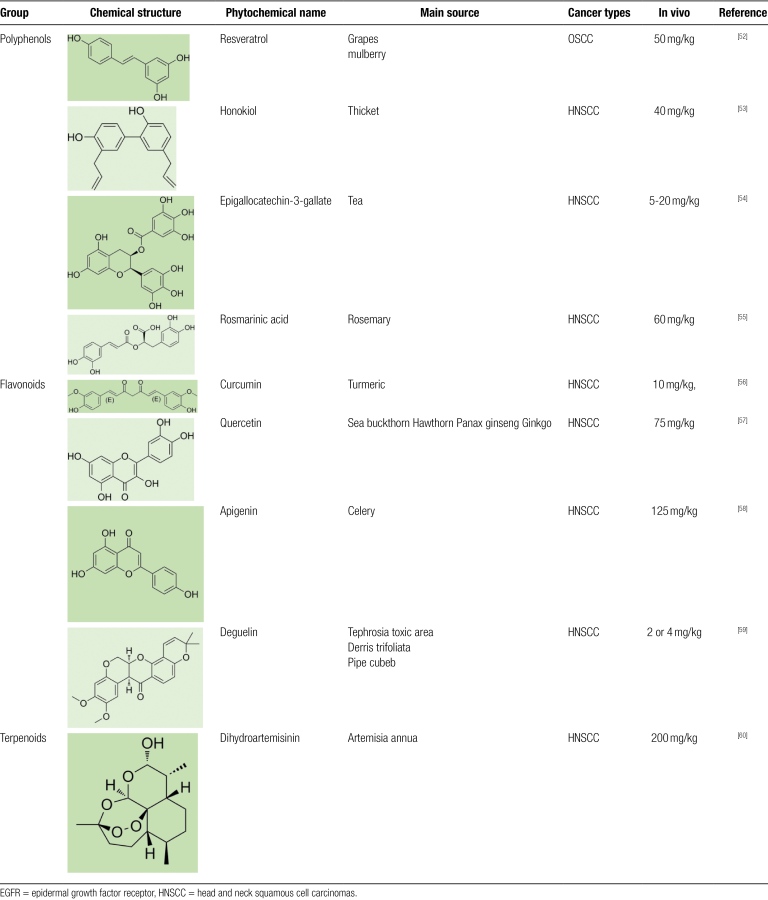
Phytochemicals targeting EGFR in HNSCC.

### 2.4. Polyphenols

Phenolic compounds are naturally occurring chemicals having a wide range of therapeutic effects.^[61]^ Natural polyphenolic chemicals can control the cell cycle, trigger apoptosis, inhibit cell adhesion, migration, proliferation, and differentiation, and have antioxidant, anti-inflammatory, anticancer, and antimutagenic properties.^[62]^ Polyphenolic substances have been demonstrated to be beneficial in the treatment of illnesses such as AIDS, heart disease, ulcers, bacterial infections, cancer, and neurological problems.^[63]^

#### 2.4.1. Resveratrol.

Resveratrol, a plant-based antioxidant found in red grapes, has anticancer and anti-inflammatory properties.^[64]^ Resveratrol has been shown to reverse multidrug resistance in cancer cells and to sensitize cancer cells to conventional chemotherapy medicines when combined with clinically utilized medications.^[65,66]^ Resveratrol effectively suppressed RCP-induced Zeb1 expression by preventing the 1 integrin endosomal cycle and EGFR activation, resulting in RCP-induced OSCC invasion suppression, revealing the importance of RCP in OSCC invasion and the reverse impact of resveratrol.^[67]^

#### 2.4.2. Honokiol.

Honokiol is a physiologically active bisphenol phytochemical with anticancer, antioxidant, anti-inflammatory, antiangiogenic, and antiangiogenic properties. Honokiol passes across the blood-brain barrier.^[68,69]^ Honokiol inhibits the viability of HNSCC cells, which induced apoptosis, and reduced the expression levels of cell cycle proteins and Cdks in HNSCC cells.^[70]^ In addition, the inhibitory effect of Honokiol on EGFR in tumor xenografts was also associated with reduced expression of mTOR and its downstream target proteins p70S6K and 4E-BP1.^[71]^

#### 2.4.3. Epigallocatechin-3-gallate (EGCG).

The principal bioactive component of green tea, EGCG, inhibits the activation of the EGFR and associated signaling pathways, as well as inhibiting cell proliferation and induces apoptosis in cancer cells.^[51,72]^ The 70% fatal dosage (IC[70]) of EGCG was found to be 10 m/mL in 2 human HNSCC cell lines, YCU-N861 and YCU-H891. Treatment with EGCG increased the proportion of cells in the G (1) phase of the cell cycle, and it inhibited EGFR, Stat3, and ERK protein phosphorylation, as well as basal and transforming growth factor-stimulated c-fos and cell cycle protein D1 promoter activity.^[73]^

#### 2.4.4. Rosmarinic acid (RA).

Numerous dietary and culinary plants and medicinal plants contain the polyphenol metabolite RA. In addition to its nutritional and therapeutic value, RA is a significant anticancer phytochemical due to its multi-targeted anticancer actions. These qualities provide a variety of therapeutic benefits for RA in addition to its traditional use as a food source.^[74]^ Blue light treatment of rosmarinic acid led to a marked reduction in cell viability, EGFR activation, and H_2_O_2_ levels in all HNSCC cell lines. By inhibiting the activation of EGFR and the production of H_2_O_2_, RA/blue light effectively reduced the proliferation of HNSCC cells.^[75]^

### 2.5. Flavonoids

Flavonoids are a type of plant secondary metabolite with polyphenolic structures that is abundant in fruits and vegetables and is an essential natural product.^[76]^ Flavonoids are categorized into the following groups based on the degree of oxidation of the 3-carbon bond (C3) structure and the position of the B-ring connection: flavones, flavonols, flavanols, flavanols or catechins, anthocyanins, and chalcones. Flavonoids are widely employed in a variety of nutritional, medicinal, and cosmetic products because of their antioxidant, anti-inflammatory, antimutagenic, and anticarcinogenic characteristics, as well as their capacity to control important cellular enzyme processes.^[77]^

#### 2.5.1. Curcumin.

Curcumin has anticancer properties and influences several biological processes involved in mutagenesis, oncogene expression, apoptosis, cell cycle control, carcinogenesis, and metastasis.^[78]^ Curcumin also affects certain growth factor receptors and cell adhesion molecules that are implicated in tumor development, angiogenesis, and metastasis. When combined with 5-FU or DOX, Curcumin decreased cell growth by downregulating the EGFR-ERK1 signaling molecules.^[79]^ When combined with cisplatin, curcumin antiproliferative effect was amplified. Inhibition of the EGFR molecular signaling cascade in mice in vivo, patient-derived xenografts, and HNSCC cell lines simultaneously slowed the growth of artificial tumors.^[80]^

#### 2.5.2. Quercetin.

Quercetin is widely found in the stem bark, flowers, leaves, buds, seeds, and fruits of many plants, mostly in the form of glycosides.^[81]^ Kun et al demonstrated that quercetin is a potent anticancer agent that inhibits the EGFR/AKT pathway in oral cancer and counteracts the metastasis of HNSCC mediated by MMP-2 and MMP-9 with high EGFR expression.^[82]^ An ERL-R5-derived xenograft mouse model confirmed the growth inhibitory efficacy of quercetin, a study reported. Furthermore, siRNA-mediated PKM2 knockdown mimicked the effects of quercetin and made ERL-R cells more sensitive to erlotinib.^[83]^ The addition of quercetin prevented the development of erlotinib-mediated resistance by enhancing apoptosis.

#### 2.5.3. Apigenin.

In nature, apigenin is widely distributed. It is prevalent in temperate zone vegetables and fruits, primarily in the Rhynchophyceae, Verbenaceae, and Curculaceae families, with celery having the highest content.^[84]^ The anticancer potential of apigenin has been demonstrated through its ability to modulate various cellular signaling pathways.^[85]^ Apigenin has been reported to dose-dependently inhibit the survival and induce apoptosis in HNSCC cells. In addition, apigenin reduced ligand-induced phosphorylation of EGFR and ErbB-2 and impaired their downstream signaling.^[86]^

#### 2.5.4. Deguelin.

Having antitumor properties, deguelin is a naturally occurring rotenone compound.^[87]^ IGF1R, AKT, and ERK1/2 constitutively phosphorylated levels decreased along with the deguelin-induced apoptosis. Both IGF-1 and epidermal growth factor-induced AKT activation were inhibited by deguelin. These findings suggested that deguelin offers a useful therapeutic approach for patients with HNSCC by demonstrating an antitumor effect by targeting AKT in dual-axis signaling pathways such as EGFR and IGF1R.^[88]^ In addition, the combination of epidermal growth factor receptor tyrosine kinase inhibitors with de-glutethimide is a potential treatment for HNSCC with PIK3CA mutations.^[89]^

### 2.6. Terpenoids

Terpenoids, also known as isoprenoids, are among the most numerous and structurally varied natural compounds found in a wide range of plants.^[90]^ Terpenoids have been proven in vitro, preclinical, and clinical investigations to have a wide variety of essential pharmacological effects in the treatment of cancer, malaria, inflammation, and numerous infectious disorders.^[91]^ Terpenoids found in nature provide new potential for the creation of low-risk medications.^[92]^

#### 2.6.1. Dihydroartemisinin.

dihydroartemisinin (DHA), a clinically useful substance that is frequently used to treat malaria, is an active metabolite of artemisinin and its derivatives. DHA has anticancer properties through several molecular mechanisms, including apoptosis induction, tumor metastasis, angiogenesis inhibition, immune system stimulation, autophagy induction, and endoplasmic reticulum stress.^[93–95]^ Osimertinib exerted synergistic cytotoxicity against FaDu and CAL27 HNSCC cells when combined with Dihydroartemisinin. This dual combination inhibited the expression of AXL. Meanwhile, the anticancer potential of the combination of osimertinib and DHA has been validated in vivo on mouse FaDu and CAL27 xenografts with no significant side effects.^[96]^

## 3. Conclusion and perspective

Recent molecular studies have made progress in identifying the alterations in the pathogenesis of HNSCC and suggest useful new molecular biomarkers for early diagnosis, individualized management, and targeted therapy. The EGFR pathway plays a key role in the development of HNSCC, and therefore targeting the EGFR pathway is a reliable approach for the treatment of HNSCC. Transmembrane protein 16A (TMEM16A) has also been found to play an important role in the development of HNSCC.^[97,98]^ Human papillomavirus (HPV) infection can regulate TMEM16A expression along with epidermal growth factor receptor (EGFR), and its phosphorylation has been described as a potential common biomarker for HPV-positive cancers.^[99]^ Considering that EGFR forms a functional complex with TMEM16A and is a common biomarker for HPV, there may be an interaction between TMEM16A expression and HPV-induced HNSCC.^[100]^ Activation of EGFR may trigger the synthesis of programmed death ligand 1 through activation of the nuclear factor kappa B pathway and JAK/intranuclear transcription factors 3 pathway.^[101]^ Combination therapy with TMEM16A and programmed death ligand 1 inhibitors may improve survival in patients with HNSCC, especially in patients resistant to anti-EGFR inhibitor therapy.^[102]^ By reaching a new level of understanding of HNSCC, the field is ready to conduct chemoprevention trials based on rigorous biologic validation for the benefit of all affected individuals.

In recent decades, phytochemicals have received much attention as potential sources for chemoprevention and treatment of a wide range of tumors. The redifferentiation effects of some compounds and their synergistic antitumor effects in combination with other therapeutic approaches have clear advantages in the treatment of HNSCC.^[103]^ Although preclinical studies have shown that many phytochemicals have all the properties required for clinical trials as adjuvants in the treatment of HNSCC, the mechanisms of their anticancer activity and their direct molecular targets are necessary for predicting effective interventions against human tumors in vivo, are not yet fully understood. This review provides an overview of the available literature and reports focusing on the in vitro effects of Phytochemicals targeting EGFR in various cellular models of HNSCC and in vivo effects in animal models, and clarifies the importance of EGFR as a current therapeutic target for HNSCC. Finally, phytochemicals have few adverse effects, but challenges remain in terms of bioavailability, toxicology, and possible drug resistance. In addition, few studies have tested their effects in vivo in mouse models. Since no clinical trials have yet been conducted to demonstrate their efficacy against HNSCC in humans, further validation of the selected secondary phytochemicals through preclinical studies is needed before the clinical application can be promoted.^[104]^ However, such compounds’ bioavailability, absorption, and effectiveness have shown promise in light of recent advancements in nanotechnology and related fields.^[105]^ Based on our review, we conclude that plant extracts targeting epidermal growth factor receptors are potentially effective candidates for the development of new drugs for the treatment of HNSCC and provide an idea for further development and application of herbal drugs for the treatment of cancer.

## Acknowledgments

The reviewers and all reference writers have been thanked by the authors.

## Author contributions

**Data curation:** Shaling Li.

**Formal analysis:** Shaling Li.

**Funding acquisition:** Yongdong Sun.

**Supervision:** Yongdong Sun.

**Validation:** Yongdong Sun.

**Writing – original draft:** Shaling Li.

**Writing – review & editing:** Shaling Li.
